# Persistent Pulmonary Hypertension of Newborns Secondary to Labile Hypoxemia Associated With Cyanosis: A Case Series

**DOI:** 10.7759/cureus.54016

**Published:** 2024-02-11

**Authors:** Anushka P Bhagwat, H V Sharath, Nikita H Seth, Saurabh N Puri

**Affiliations:** 1 Department of Paediatric Physiotherapy, Ravi Nair Physiotherapy College, Datta Meghe Institute of Higher Education and Research, Wardha, IND; 2 Department of Neurophysiotherapy, Ravi Nair Physiotherapy College, Datta Meghe Institute of Higher Education and Research, Wardha, IND

**Keywords:** bronchopulmonary dysplasia, nrds, high-risk neonates, chest physiotherapy, pphn

## Abstract

Persistent pulmonary hypertension of the newborn (PPHN) is a condition that can be fatal, marked by increased pulmonary vascular resistance that causes blood to shunt from the right to the left. Six infants that present with PPHN due to labile hypoxemia and related cyanosis are examined in this case series. Clinical manifestations, such as premature deliveries, maternal problems, and different reactions to early therapies, are revealed by perinatal and postnatal histories. The newborns' respiratory distress prompted the use of oxygen supplementation and continuous positive airway pressure (CPAP), but intubation was required due to continued hypoxemia. The series aims to establish a way for further study in this crucial area while offering insightful contributions to the clinical subtleties of PPHN and illustrating the importance of specific therapeutic approaches.

## Introduction

A persistent rise in pulmonary vascular resistance following delivery leads to poor oxygenation, which is the hallmark of the clinical illness known as persistent pulmonary hypertension of the newborn (PPHN). PPHN was defined based on a combination of clinical and echo-cardio-graphical characteristics [1]. The prevalence of PPHN in neonates varies from 0.4 to 6.8 per 1000 live births [2-3]. The majority of PPHN is caused by maladaptation of the normally established pulmonary vasculature due to an imbalance of vasoactive substrates, which frequently results from sepsis, pneumonia, meconium aspiration syndrome, or hypoxia. The pulmonary vasculature's abnormal development, or maldevelopment, is primarily idiopathic but can also be linked to fetal anemia, persistent hypoxia, or early ductus arteriosus closure. Prolonged hypoxemia, perinatal ischemia damage, or an underlying lung parenchymal disease process are some of the causes of the pulmonary artery constriction observed in PPHN [4,5].

Hypoxemia results from a right to the left shunt of deoxygenated blood via the persistent patent foramen ovale (PFO) or patent ductus arteriosus (PDA) when the pulmonary vascular resistance is greater than the systemic vascular resistance [6]. Nitric oxide helps term and near-term newborns with hypoxic respiratory failure requiring less extracorporeal membrane oxygenation (ECMO). Nevertheless, over 40% of near-term newborns receiving INO treatment either pass away or still need ECMO [7]. Fetal pulmonary hypertension, or PPHN, persists when the typical newborn circulatory transition is disrupted, which prevents the condition from being resolved. Less blood reaches the lungs when the pulmonary pressure is high. Cyanosis is caused by a mismatch in ventilation-perfusion (VQ) and extrapulmonary right-to-left shunting of deoxygenated blood across the PDA and PFO [8]. The pulmonary artery to the aorta shunt via the PDA causes differential cyanosis, which is characterized by saturation in the lower limb that is 5-10% lower than that in the right upper limb. The degree of cyanosis in the upper and lower limbs is comparable if the PDA is closed and the shunt is just at the PFO level. PPHN is characterized by labile hypoxemia, which is a dramatic shift in oxygen saturation with little to no change in ventilator settings. This is caused by a change in the volume of the right-to-left shunt as a result of minute adjustments to the delicate balance between PVR and SVR [9,10].

Neonates delivered at or near term (at gestational age > 34 weeks) may have hypoxic respiratory failure due to a variety of problems, including congenital diaphragmatic hernia (CDH), pneumonia or sepsis, aspiration of meconium, respiratory distress syndrome, and primary persistent pulmonary hypertension [11]. In addition to ECMO, conventional treatment includes oxygen support, mechanical breathing, alkalosis induction, neuromuscular blockade, and sedation. It has not been discovered that any of these treatments lowers mortality or eliminates the requirement for ECMO [12,13]. The mortality rate of infants with PPHN is approximately 10% but is higher in infants with underlying conditions, such as CDH. Up to 25% of infants with PPHN will have significant neurodevelopmental impairment at two years of age [14,15]. When associated with labile hypoxemia and cyanosis, the condition becomes even more challenging. Physiotherapy and occupational therapy play crucial roles in managing patients with PPHN, and their inclusion in the case series can be justified for several reasons.

Respiratory management is done by physiotherapists, and it will assist in optimizing ventilation strategies, including the use of non-invasive ventilation or mechanical ventilation as required. Breathing exercises such as deep breathing exercises, diaphragmatic breathing, and controlled breathing can be employed to enhance respiratory function. Patients with secretion clearance in PPHN may experience difficulties in clearing respiratory secretions. Chest physiotherapy techniques, such as percussion and vibration, can help in mobilizing and clearing secretions, reducing the risk of respiratory complications.

Positioning and handling can assist in positioning the newborn to optimize lung expansion and improve oxygenation. Proper positioning can also prevent complications such as pressure sores and musculoskeletal issues, and sensory stimulation techniques can be employed to promote neurodevelopment and enhance the infant's responsiveness to external stimuli, which is important for overall development. Collaborative care for PPHN and a multidisciplinary approach involving physiotherapists, occupational therapists, neonatologists, and other healthcare professionals ensure comprehensive and coordinated care for these newborns. Including physiotherapy and occupational therapy in a case series on PPHN provides valuable insights into the holistic care of these newborns. It emphasizes the importance of addressing both respiratory and developmental aspects to improve outcomes and enhance the quality of life for these infants and their families.

## Case presentation

Case 1

An 860 g female baby was born at 29 weeks via preterm normal vaginal delivery in the month of July 2023 in a private hospital in Amravati, India. The baby did not cry immediately after birth and had a case of respiratory distress and extremely low birth weight, and the preterm baby was referred to AVBRH for further management. Here, the baby was admitted to the NICU where she was on continuous positive airway pressure (CPAP). On examination, it was seen that primitive reflexes were present. Due to harsh bronchial breathing on chest auscultation, the baby was referred to pediatric physiotherapy for further management.

Perinatal history: A pre-term female infant was delivered via spontaneous vaginal delivery. The pregnancy was uneventul, and the mother had regular prenatal checkups. No known risk factors or complications were identified during the antenatal period.

Postnatal history: The neonate baby did not cry immediately after birth, and the Apgar scores were 9 and 9 at one and five minutes, respectively. However, within the first hour, the infant exhibited labile hypoxemia associated with cyanosis. Despite initial attempts with CPAP and oxygen supplementation, the neonate's condition worsened, leading to the diagnosis of PPHN.

Case 2

A female infant was delivered by cesarean section at 34 weeks of gestation, with a birth weight of 2.475 g, head circumference of 30 cm, and length of 43 cm. Her Apgar score was 8 at one minute and 8 at five minutes, and she screamed right away after birth. At 15 minutes of age, the infant was intubated and given positive pressure ventilation due to poor oxygen saturation, significant retractions, and grunting even with a 100% fraction of inspired oxygen (FiO2) and CPAP applied. After the infant was sent to the neonatal intensive care unit (NICU), contact isolation and flight safety measures were taken, and then the baby was referred to pediatric physiotherapy for further management.

Perinatal history: Because of the mother's hypertension, an emergency cesarean section was performed to deliver a premature girl newborn at 34 weeks of gestation. Proteinuria and hypertension were symptoms of the pregnancy. The purpose of antenatal corticosteroids was to improve fetal lung maturity.

Postnatal history: The baby had retractions and grunting as soon as it was born due to respiratory difficulties. Oxygen treatment and CPAP were ineffective in treating labile hypoxemia. After the baby was intubated at the age of 15 minutes, further testing identified PPHN as the underlying reason.

Case 3

A 32-week pre-term male infant delivered via normal vaginal delivery presented with a birth weight of 2.5 kg, head circumference of 33 cm, and length of 48 cm. However, at 10 minutes of age, the infant developed sudden-onset stridor and respiratory distress. CPAP and FiO2 of 50% were initiated, but due to worsening respiratory distress and high-grade retractions, intubation was performed. The baby was shifted to the NICU, with the precaution of airborne and contact isolation, and then the baby was referred to pearlitic-physiotherapy for further management.

Perinatal history: A spontaneous vaginal delivery resulted in the birth of a pre-term male child. The woman underwent routine prenatal checkups, and the pregnancy progressed without incident. During the prenatal phase, no recognized risk factors or problems were found.

Postnatal history: The baby cried as soon as it was born, and at one and five minutes, the Apgar scores were 9 and 9, respectively. However, the baby showed signs of cyanosis and labile hypoxemia during the first hour. PPHN was diagnosed when the neonate's health deteriorated despite early attempts with CPAP and oxygen supplementation.

Case 4

A pre-term male infant born at 32 weeks of gestation via spontaneous vaginal delivery presented with a birth weight of 1.8 kg, head circumference of 28 cm, and length of 40 cm. Immediate crying was followed by respiratory distress, and the Apgar scores were 7 and 8 at one minute and five minutes, respectively. CPAP and initial respiratory support were provided; however, the infant's oxygenation remained labile. Intubation was performed at 25 minutes of age due to worsening respiratory distress. The baby was shifted to the NICU, with the precaution of airborne and contact isolation, and then the baby was referred to pediatric physiotherapy for further management.

Perinatal history: An elective cesarean section was used to deliver a pre-term male child. There were no recognized risk factors, and the pregnancy was straightforward. Prenatal ultrasounds revealed no abnormalities.

Postnatal history: The newborn seemed well at first, but within the first hour, cyanosis, labile hypoxemia, and respiratory distress occurred. The neonate's oxygen saturation remained unstable despite CPAP and high-flow oxygen therapies, which led to the diagnosis of PPHN. 

Case 5

A term female infant delivered by elective cesarean section presented with a birth weight of 3.8 kg, head circumference of 34 cm, and length of 50 cm. Initial crying and Apgar scores of 9 and 9 at one minute and five minutes, respectively, were noted. However, at 30 minutes of age, the infant exhibited sudden onset cyanosis and desaturation. Despite initial CPAP and FiO2 of 60%, the respiratory distress worsened, leading to intubation.

Perinatal history: Because of fetal discomfort, an emergency cesarean section was performed to deliver a term female newborn. Gestational diabetes complicated the pregnancy, although it was controlled with nutrition.

Postnatal history: Soon after delivery, the baby showed symptoms of respiratory distress, including cyanosis and labile hypoxemia. Inadequate initial CPAP and oxygen control resulted in intubation. PPHN was identified during the evaluation as the main reason for the respiratory impairment.

Case 6

A female infant delivered by cesarean section at 34 weeks of gestation presented with a birth weight of 2.475 g, head circumference of 30 cm, and length of 43 cm. Immediate crying was noted after birth, and the Apgar scores were 8 and 8 at one minute and five minutes, respectively. At 15 minutes of age, the infant was intubated due to severe respiratory distress characterized by grunting, significant retractions, and low oxygen saturation, despite initial management with CPAP and a FiO2 set at 100%. Following intubation, positive pressure ventilation was initiated to address the escalating respiratory distress. Despite these interventions, the infant's oxygenation status remained suboptimal. The decision was made to transfer the neonate to the NICU for further evaluation and management. Precautions were taken with airborne and contact isolation measures due to the clinical presentation and the need for close monitoring.

Perinatal history: Prolonged second-stage labor resulted in a vacuum extraction delivery of a pre-term female child. There were no known risk factors, and the pregnancy was uneventful.

Postnatal history: The baby screamed when it was born, but within 20 minutes, breathing difficulties set up. The infant's oxygen saturation remained unstable despite attempts to treat it with CPAP and oxygen treatment, which resulted in intubation. The diagnosis of PPHN was then made using echocardiography.

On examination

The cardiorespiratory examination in neonates is a crucial part of the newborn assessment, ensuring the early detection of any potential issues with the cardiovascular and respiratory systems. Here is a general outline of what a healthcare professional might assess during a cardiorespiratory examination in neonates, which is noted in Table 1.

**Table 1:** Cardiopulmonary examination

Cardiopulmonary Examination	Case 1	Case 2	Case 3	Case 4	Case 5	Case 6
Types of Breathing	Slightly increased respiratory rate with mild tachypnoea	Increased respiratory rate with moderate retractions	Rapid and laboured breathing with severe retractions	Rapid, shallow breaths during hypoxic spells	Reduced respiratory rate and effort with improvement in oxygenation.	Steady, normal respiratory rate after successful treatment
Symmetry of Chest	Bilaterally symmetrical chest expansion	Asymmetric chest expansion with increased work of breathing	Markedly asymmetric chest expansion with intercostal retractions	Uneven chest expansion, especially during spells	Improved chest expansion and reduced retractions	Symmetrical chest expansion, no signs of respiratory distress
I:E Ratio	Normal inspiratory to expiratory ratio	Prolonged inspiratory phase, suggestive of increased effort	Pronounced prolonged inspiratory phase, indicative of severe respiratory distress	Variable, with prolonged inspiration during hypoxic events	Gradual normalization of inspiratory to expiratory ratio	Return to a normal inspiratory to expiratory ratio post-intervention

The neuro-muscular examination in neonates is an essential part of the newborn assessment to evaluate the baby's neurological and muscular development. Here is a general outline of what a healthcare professional might assess during a neuro-muscular examination in neonates, which is noted in Table 2.

**Table 2:** Neurological stimulation

Primitive Reflexes	Case 1	Case 2	Case 3	Case 4	Case 5	Case 6
Palmar Reflex	Present	Absent	Present	Present	Absent	Present
Plantar Reflex	Present	Present	Present	Absent	Present	Present
Sucking Reflex	Absent	Present	Present	Absent	Present	Present
Rooting Reflex	Present	Absent	Present	Present	Present	Absent

Rehabilitation protocol 

**Figure 1:**
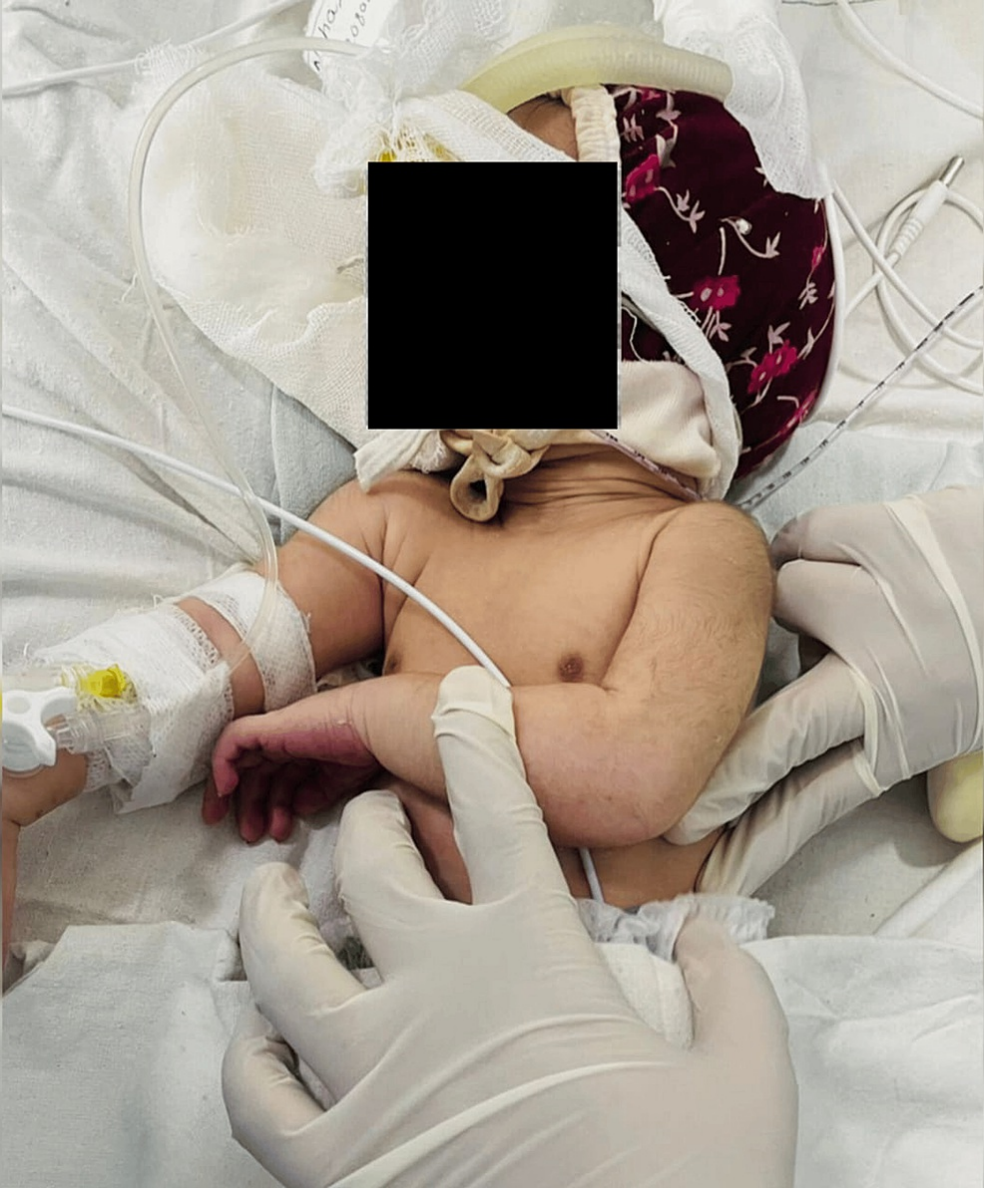
Vibration for the different lobes

**Figure 2:**
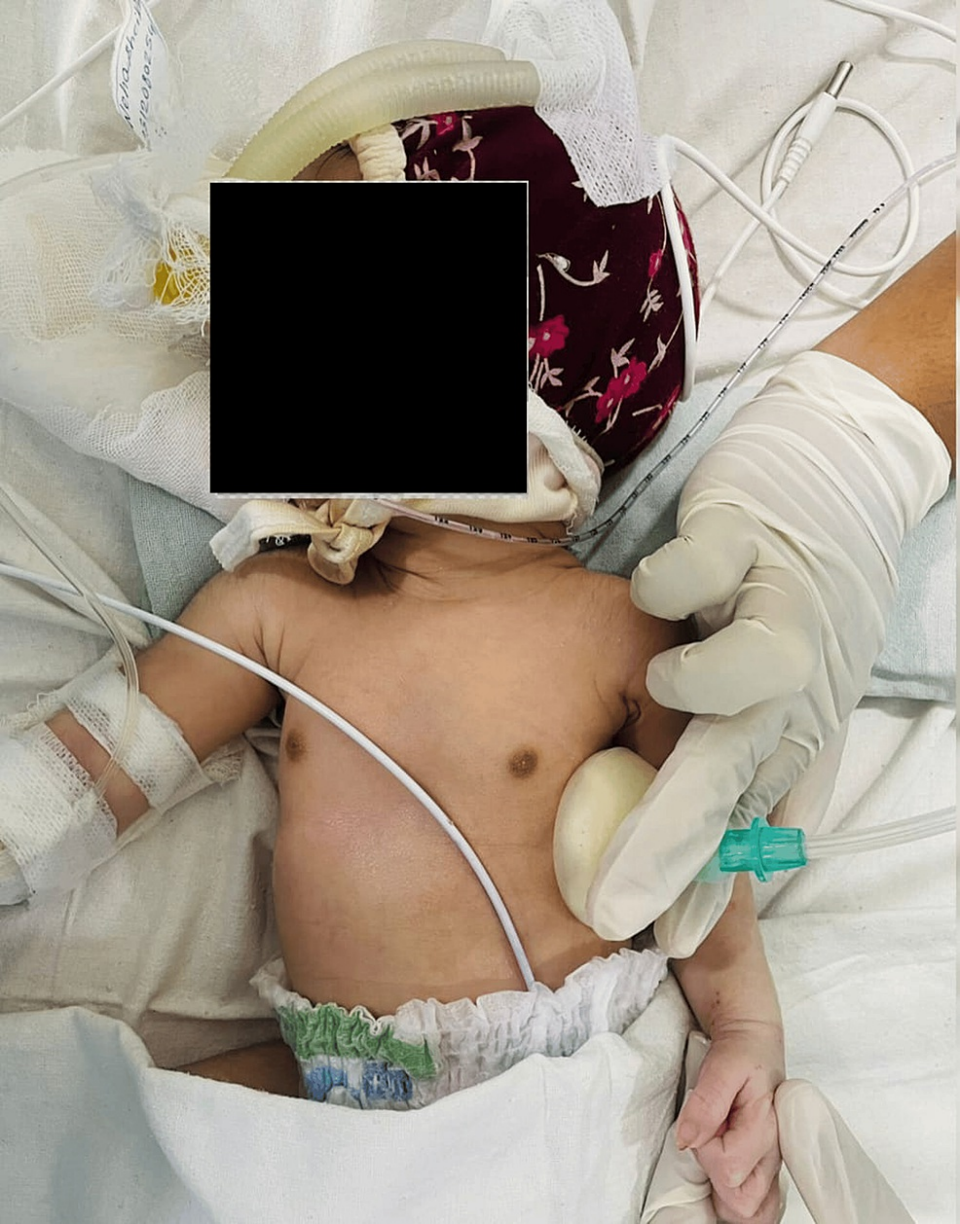
Percussion with percussion cup

**Figure 3:**
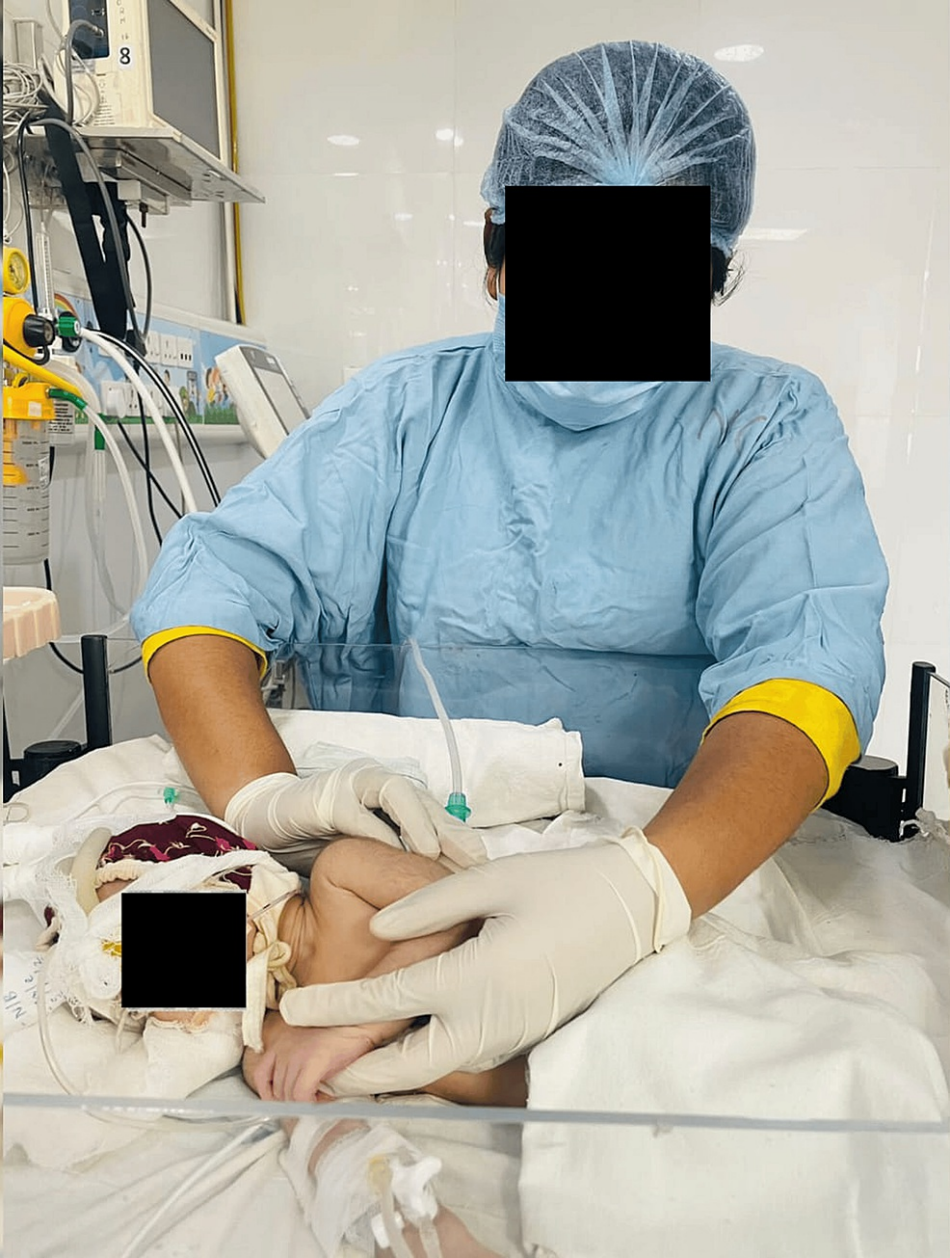
Vojta method with percussion

The pediatric physiotherapist conducted a comprehensive assessment, focusing on respiratory mechanics, chest wall movement, and overall respiratory muscle function. Specific attention was given to addressing the severe retractions noted during the initial presentation. Therapeutic interventions, including chest physiotherapy techniques and respiratory exercises, were initiated to optimize ventilation and improve lung compliance. Simultaneously, we continued to monitor the infant's vital signs, blood gas values, and overall clinical status. The description of Figures 1-3 is given in Table 3.

**Table 3:** Rehabilitation protocol

TREATMENT	DESCRIPTION	INTENSITY
Percussion	Chest percussion involves tapping the chest using percussor cups, resembling suction cups, to create vibrations in the airways of the lungs. These vibrations aid in loosening mucus, facilitating its removal through coughing, as shown in Figure 2.	30 percussion for 5 sets
Active Gentle Vibration	A rapid, sensitive trill-type movement was used to actively deliver gentle vibrations while exhaling. Mild vibrations were applied after percussion to help shift secretions in the direction of the bigger airways. Each newborn's chest vibrations were manually applied by covering the targeted region with one hand's fingers on the chest wall. The isometric contraction of the hand and forearm muscles produced a faint vibrating motion. Concurrently, the other hand supported the head of the infant, keeping the palm cupped to cradle the head for the length of the treatment shown in Figure 1.	30 vibrations for 5 sets
Vojta Method	The Vojta method involves specific reflex stimulation and movement patterns to promote motor development and improve muscle function. It is commonly used in treating infants and young children with neurological or motor disorders. The method is based on the idea that certain reflexes and movement patterns can activate and integrate the central nervous system, leading to improved motor control. In the context of neonates (newborns), the Vojta method may be employed to address issues related to muscle tone, coordination, and overall motor development. However, it's essential to note that any therapeutic approach, including the Vojta method, should be administered by trained and qualified healthcare professionals, such as physical therapists or pediatricians. If you have a specific question about the Vojta method in neonatal care or if you meant something else by "Vojta in neonates," please provide more details so I can offer a more precise and helpful response shown in Figure 3.	Immediately after the percussion and active gentle vibration

Outcome measures

Tables 4-5 show the outcome measure, which was taken before and after the physiotherapy intervention.

**Table 4:** Outcome measure before treatment APGAR: Appearance, pulse, grimace, activity and respiration; ABG: Arterial blood gas; PCO2: Partial pressure of carbon dioxide; PO2: Partial pressure of oxygen; pH: Potential of hydrogen; kg: kilogram; gm: gram

SR NO.	Outcome measure(pre)	Case 1	Case 2	Case 3	Case 4	Case 5	Case 6
1.	APGAR	8 - 1 minute, 9 - 5 minutes	6 - 1 minute, 8 - 5 minutes	5 - 1 minute, 8 - 5 minutes	4 - 1 minute, 7 - 5 minutes	7 - 1 minute, 9 - 5 minutes	9 - 1 minute, 9 - 5 minutes
2.	Neonatal behavioural assessment scale	35	28	20	30	38	40
3.	Breastfeeding scale	7/10	5/10	3/10	6/10	8/10	9/10
4.	Ballard score	16	16	17	16	18	19
5.	Weight of the baby	860 gm	2.47 kg	3.5 kg	1.8 kg	3.8 kg	2.475 kg
6.	ABG report - investigation	pH 7.35, pO2 65 mmHg, pCO2 45 mmHg	pH 7.28, pO2 55 mmHg, pCO2 50 mmHg	pH 7.20, pO2 40 mmHg, pCO2 60 mmHg	pH 7.25, pO2 50 mmHg, pCO2 55 mmHg	pH 7.40, pO2 70 mmHg, pCO2 40 mmHg	pH 7.45, pO2 80 mmHg, pCO2 35 mmHg

**Table 5:** Outcome measure after treatment ABG: Arterial blood gas; PCO2: Partial pressure of carbon dioxide; PO2: Partial pressure of oxygen; pH: Potential of hydrogen; kg: kilogram

SR NO.	Outcome measure(post)	Case 1	Case 2	Case 3	Case 4	Case 5	Case 6
1.	Neonatal behavioural assessment scale	38	32	25	35	40	45
2.	Breastfeeding scale	8/10	6/10	4/10	7/10	9/10	10/10
3.	Ballard score	18	17	16	17	18	19
4.	Weight of the baby	1.5 kg	3.5 kg	3.8 kg	2.75 kg	3.8 kg	3 kg
5.	ABG report - investigation	pH 7.40, pO2 75 mmHg, pCO2 42 mmHg	pH 7.38, pO2 65 mmHg, pCO2 48 mmHg	pH 7.35, pO2 50 mmHg, pCO2 55 mmHg	pH 7.38, pO2 60 mmHg, pCO2 50 mmHg	pH 7.45, pO2 80 mmHg, pCO2 35 mmHg	pH 7.48, pO2 85 mmHg, pCO2 32 mmHg

## Discussion

The case series on PPHN secondary to labile hypoxemia associated with cyanosis sheds light on a critical and complex clinical scenario in neonatology. This discussion aims to explore various aspects of the case series, including the underlying pathophysiology, clinical manifestations, diagnostic challenges, and the implications for management and prognosis. First and foremost, the case series emphasizes the significant association between labile hypoxemia, characterized by fluctuating oxygen levels, and the development of PPHN. The pathophysiological mechanisms leading to persistent pulmonary hypertension in the context of labile hypoxemia and cyanosis involve impaired oxygen exchange in the lungs, leading to sustained pulmonary vasoconstriction. Understanding this intricate relationship is crucial for clinicians to recognize and address the condition promptly.

The clinical manifestations highlighted in the case series likely encompass a spectrum of symptoms ranging from respiratory distress, cyanosis, and labored breathing to more severe complications, such as hypotension and organ dysfunction. The variability in clinical presentation underscores the importance of a high index of suspicion, especially in neonates with risk factors such as perinatal asphyxia or respiratory distress syndrome. Diagnosing PPHN secondary to labile hypoxemia can be challenging, and the case series emphasizes the need for a comprehensive diagnostic approach. This may include echocardiography to assess pulmonary artery pressures, oxygen saturation monitoring, and blood gas analysis. The recognition of cyanosis as a potential marker for labile hypoxemia and subsequent PPHN is crucial, guiding clinicians in their diagnostic workup.

The implications for management underscore the necessity of a multidisciplinary approach in caring for newborns with PPHN. Initiating appropriate respiratory support, such as mechanical ventilation or high-frequency oscillatory ventilation, becomes paramount in stabilizing oxygen levels and improving pulmonary blood flow. In severe cases, consideration may be given to the use of pulmonary vasodilators, although their efficacy and safety in newborns need careful evaluation. The case series also prompts discussions regarding the long-term prognosis and potential neurodevelopmental outcomes for infants affected by PPHN secondary to labile hypoxemia. The impact on neurodevelopmental outcomes may vary, necessitating ongoing monitoring and intervention from neurodevelopmental specialists to optimize long-term outcomes.

Furthermore, the case series raises ethical considerations related to decision-making in the neonatal intensive care setting. Discussions about goals of care, potential interventions, and involvement of parents in the decision-making process become crucial components of ethical care for these vulnerable newborns. A right-to-left shunt of deoxygenated blood with hypoxemia results from vasoconstriction of the pulmonary arteries in PPHN when pulmonary vascular resistance (PVR) is greater than systemic vascular resistance (SVR) [16]. This case report underscores the integral role of physiotherapy rehabilitation, with a specific focus on percussion, active gentle vibration, and the Vojta method, in the management of PPHN secondary to labile hypoxemia and associated cyanosis.

Oromotor stimulation emerges as a key component in addressing the challenges of feeding difficulties commonly observed in neonates with PPHN due to compromised respiratory function. By incorporating non-nutritive sucking exercises and gradually progressing to nutritive sucking, stimulation aids in the development of essential skills for successful feeding and contributes to improved respiratory coordination [17,18]. The case underscores the need for a tailored and multidisciplinary approach, involving physiotherapists, neonatologists, and other healthcare professionals, to optimize outcomes in these vulnerable infants [19]. Physiotherapeutic rehabilitation, encompassing chest physiotherapy and optimal positioning, addresses the respiratory challenges associated with PPHN. Research supports the role of chest physiotherapy in promoting lung expansion and secretion clearance, thereby potentially reducing episodes of labile hypoxemia [20]. Additionally, optimal positioning techniques contribute to improved respiratory mechanics, alleviating the strain on the compromised pulmonary system [21].

In conclusion, the case series on PPHN secondary to labile hypoxemia associated with cyanosis highlights the intricate interplay between oxygenation instability and pulmonary hypertension in newborns. The multifaceted nature of the condition demands a comprehensive diagnostic and therapeutic approach, involving a coordinated effort from various healthcare disciplines. Understanding the implications for management, prognosis, and ethical considerations is essential for providing optimal care to neonates facing this challenging clinical scenario.

## Conclusions

In conclusion, physiotherapy rehabilitation is essential for the management of postnatal hypoxemia, especially when cyanosis and labile hypoxemia are present. The combination of active moderate vibrations, the Vojta technique, and percussion provides a multimodal strategy to manage respiratory issues in these newborns. Enhancing lung expansion, maximizing ventilation-perfusion matching, and enhancing general respiratory muscle performance are the goals of these approaches. However, with the severity of PPHN and related clinical conditions, it is crucial to differentiate the therapies based on the unique demands and responses of each infant. For the purpose of managing PPHN, more investigation and clinical studies are necessary to determine the safety and effectiveness of various physiotherapy techniques.
